# Real-Time Megapixel Electro-Optical Imaging of THz Beams with Probe Power Normalization

**DOI:** 10.3390/s22124482

**Published:** 2022-06-14

**Authors:** François Blanchard, Takashi Arikawa, Koichiro Tanaka

**Affiliations:** 1 Département de Génie Électrique, École de Technologie Supérieure (ÉTS), Montréal, QC H3C 1K3, Canada; 2 Department of Physics, Kyoto University, Kyoto 606-8502, Japan; arikawa@scphys.kyoto-u.ac.jp (T.A.); kochan@scphys.kyoto-u.ac.jp (K.T.)

**Keywords:** terahertz, imaging, microscopy, ultrafast, electro-optic, real-time

## Abstract

In this work, we present a simple method to improve the spatial uniformity of two-dimensional electro-optical imaging of terahertz (THz) beams. In this system, near-field THz images are captured by fully illuminating a sample using conventional optical microscope objectives. Unfortunately, due to the linear relationship between the optical probe power and the measured THz electric field, any spatial variation in probe intensity translates directly into a variation of the recorded THz electric field. Using a single normalized background frame information map as a calibration tool prior to recording a sequence of THz images, we show a full recovery of a two-dimensional flat field for various combinations of magnification factors. Our results suggest that the implementation of dynamic intensity profile correction is a promising avenue for real-time electro-optical imaging of THz beams.

## 1. Introduction

For over two decades, research into THz wave imaging has been on a constantly evolving path [1,2,3]. As methods improve, so do the related applications, such as advanced detection [4], security screening [5], plasmonic imaging [6], and potentially, communications [7]. Among imaging demonstrations, two-dimensional electro-optical imaging of THz beams, first demonstrated in the mid-1990s [8], is an important method for capturing streaming images in the time domain. This method has undergone several improvements over the years, including two-dimensional birefringence correction to smooth out imperfections in the EO crystal [9,10], dynamic background subtraction to reduce noise [11], access to the near-field region with a thin EO crystal [12], and improved sensitivity and resolution through spectral filtering of the probe light [13], to name a few.

To date, two-dimensional electro-optical imaging of THz beams has not yet been widely adopted in most THz research laboratories, which, to a certain extent, is somewhat attributable to the commercial unavailability and relative complexity of such systems. To image THz waves more simply, raster scanning approaches [1] have undergone significant advances with demonstrations of fast acquisition [14] and extremely high resolutions, down to the nanometer [15]. The above-mentioned measurement is interesting because it is compatible with commercially available THz spectrometers and mainly requires moving the sample in the THz beam path. Incidentally, this attribute has significantly stimulated the development of compression algorithms for single-pixel imaging solutions [16], whereas recent years have seen a slower improvement trend for two-dimensional EO imaging solutions. Nevertheless, to obtain real-time images of mm^2^ samples with micron-level resolution [13], two-dimensional EO imaging of THz beams continues to be an indispensable tool.

In this work, we propose the use of a single background frame to homogenize the two-dimensional EO response of time-resolved near-field THz images recorded at video frame rates. We compare our results for different THz beam illumination conditions, including 2×, 5×, 10×, and 20× magnification factors. Using a THz fractal metasurface as a testbed, we visually confirm the importance of our calibration procedure over simply performing dynamic background subtraction to recover highly resolved THz images and movies in real time. This demonstration clearly suggests the addition of this operation to the image acquisition sequence.

## 2. Materials and Methods

**Figure 1:**
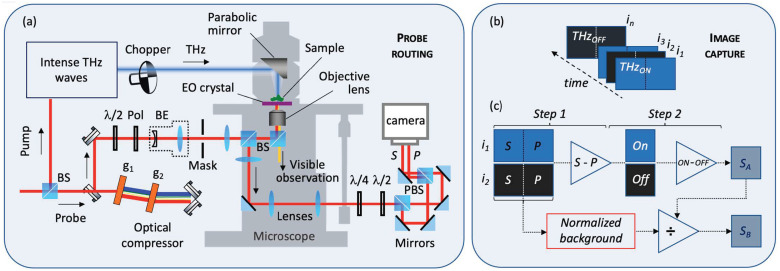
(**a**) Probing beam routing for two-dimensional THz field measurement in the near field. (**b**) Synchronization of the acquisition sequence for dynamic background subtraction. (**c**) Subtraction operation between vertical *S* and horizontal *P* images followed by dynamic background subtraction (On-Off images). The lower part of Figure (**c**) shows the additional normalization operation to the acquisition sequence. PBS: polarized beam splitter, BS: beam splitter, Pol: polarizer, BE: beam expander, g: grating, *S*: S-polarized image, *P*: P-polarized image, and *i_n_*: image number *n*.

The system used in this experiment was first reported over ten years ago [12]. Set around an intense THz pulsed source with a center frequency of about 0.7 THz [17], as illustrated in Figure 1a, the observation unit was coupled to a visible optical microscope (model FV1000 from Olympus) that easily allows changes of objective lenses. 1920 × 1080-pixel images with 16-bit resolution are captured at 50 frames per second (fps) from an sCMOS camera (model Edge 5.5 from PCO). The capture was synchronized with the laser repetition rate and the mechanical chopper to provide dynamic background subtraction [11] from an alternative sequence of THz_ON_/THz_OFF_ images, as depicted in Figure 1b. The camera has a special trigger function known as the global shutter mode, which reads the data for all pixels in parallel. As shown on the right side of Figure 1a, the probe optical beam was split into two parts on the camera to simultaneously record horizontally (*P*) and vertically (*S*) polarized images. As detailed previously [12] and illustrated in Figure 1b,c, the typical measurement is performed in two steps: first, there is the subtraction between the corresponding pixels of the *S* and *P* polarized images that have been spatially separated on the camera, followed by a background subtraction from the next image capture (i.e., when the mechanical chopper blocks the THz signal). The result of this operation corresponds to the block *S_A_* in Figure 1c.

A CCD camera was used to perform EO detection calls for certain precautions, such as avoiding the presence of saturated pixels. To control the spatial uniformity of the probe light, a 3× beam expander is sufficient to select the central portion of a Gaussian distributed beam to produce a hat-shaped illumination. Unfortunately, this system involves the use of several optics in the probing beam path. In addition to the optics shown in Figure 1a, over ten routing mirrors, two transmissive filters, and two optical delay lines can be added. The surfaces of these optics can by no means be maintained perfectly to avoid dust accumulation during daily operations. This is also especially true for objective lenses, which can easily sustain minor damage from the improper use of intense femtosecond laser probing pulses; an example of such use is focusing inside the objective lens. These tiny optical damages and small dust easily introduce probe power variations in the order of ±20% or more at the detection position. These unavoidable variations considerably affect the quality of THz images, particularly those obtained in the near field and at high magnification.

To date, the non-uniformity of the probe beam intensity has generally been mitigated by using normalized information in the frequency domain. This procedure is very effective when access exists, allowing for the recording of two sets of temporal THz images (named movie from now on), including a reference movie and a signal movie. Normalization is applied in the frequency domain by dividing the amplitude of each frequency map and subtracting the phase information between the maps in order to recover transmission and absorption parameters [12,13]. It has been shown that this method is also effective when working with nonlinear THz images to improve the near-field resolution and contrast [18]. However, frequency domain normalization is not a dynamic procedure and only applies in the case where the user records two data sets (reference and signal). It is important to note that the above-mentioned normalization cannot provide artifact-free electric field recovery for a single data set (since no normalization information is available). Therefore, recording a true uncompressed temporal THz movie without spatial distortion due to the probing light beam can be essential when working with temporal information, especially if the absolute value of the THz electric field amplitude distribution is paramount for the analysis.

## 3. Results

**Figure 2:**
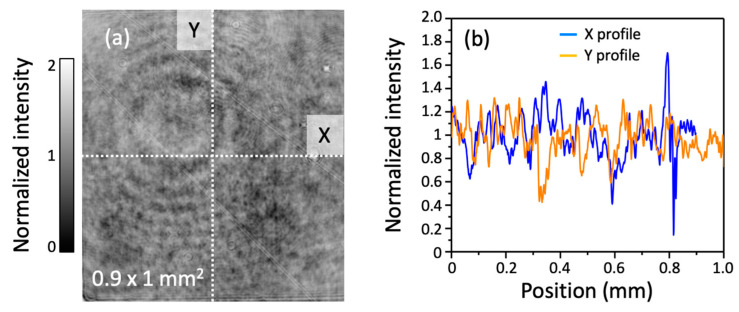
(**a**) Normalized 2D profile of the background probe intensity with a 5× objective lens, i.e., without terahertz field applied at the sensor position. (**b**) Profiles extracted in the horizontal (blue) and vertical (orange) directions at the positions shown by the dotted lines in (**a**).

In Figure 2a, we show a typical 2D map of a background frame information captured with a 5× objective lens. This 2D map was normalized by the mean value of all pixels included in that image. For a perfect hat-shaped image, a flat profile centered around 1 should be obtained. However, after taking two profiles extracted in the horizontal and vertical directions, as shown respectively by the blue and orange curves in Figure 2b, significant variations of the probe beam could be observed, with some pixels exceeding 70% of the normalized value. As previously described, the origin of the fringe patterns comes from the diffraction caused by each of the dust particles encountered in the probe’s path. The size and superposition of these fringe patterns depend respectively on the distance between the dust and the image plane of the camera, and their position on the optical beam. 

For a balanced imaging scheme, Equation (1) provides the relation between the probe power and the detected THz field via the induced birefringence in a LiNbO_3_ (LN) EO crystal, given by [19]:

(1) $$\frac{A-B}{A+B}=sin\left(\theta \right)\approx \frac{2\pi }{\lambda }n_{e}^{3}r_{33}E_{THz}L$$

where *A* and *B* are the probe power read on both photodiodes, sinθ is the induced modulation, $n_{e}^{3}$ is the refractive index, $r_{33}$ is the electro-optic coefficient, *L* is the EO crystal thickness, and *E* is the THz electric field value.

In Equation (1), it can be seen that the amount of probing light, i.e., the term (*A* + *B*), has a linear relationship with the electric field evaluation. In contrast to a previous work by Z. Jiang et al. [9], where a square root normalization of the background signal was considered and which takes into account their special unit of polarization analysis, our balanced imaging scheme requires only a linear correction, i.e., a correction proportional to the probe power, as explicitly defined by Equation (1). To create a normalization background frame *N_bck_*, we used the first recorded background frame and performed the following operation:

(2) $$N_{bck}=\frac{S_{i}+P_{i}}{\frac{1}{n}\sum_{i=1}^{n}\left(S_{i}+P_{i}\right)}$$

where the numerator gives the matrix sum of the two-dimensional background images for vertical (*S_i_*) and horizontal (*P_i_*) polarization, and *n* is the total number of pixels for an image, e.g., the size of (*P_i_*). Simply stated, the denominator represents the average (scalar) value of the numerator.

**Figure 3:**
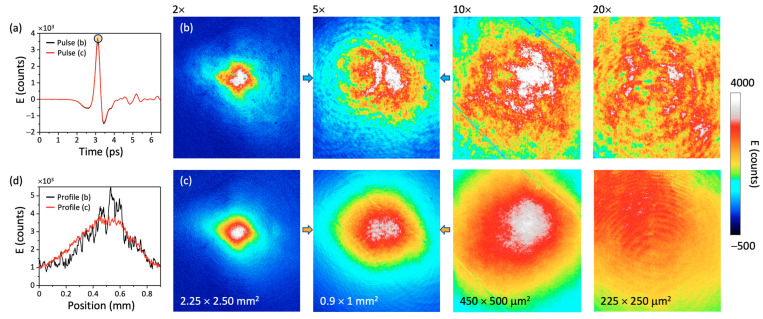
(**a**) Time profile (extracted from 10 pixels) of the THz pulse used for this experiment. The 2D maps in (**b**) represent the THz electric field at the peak position, i.e., indicated by a yellow dot in (**a**), for the 2×, 5×, 10×, and 20× objectives without probe power normalization, respectively, whereas the maps in (**c**) show the improvement after applying a background probe normalization. (**d**) Extraction of temporal beam profiles using a 5× objective lens without (black) and with (red) probe normalization at the positions between the arrows in 5× maps of figures (**b**,**c**), respectively. All THz images are composed of a cropped area of 834 × 960 pixels.

Figure 3 presents a comparison of THz imaging using standard dynamic background subtraction versus dynamic background subtraction, followed by a probe power normalization frame *N_bck_*, which corresponds to a comparison between cases *S_A_* and *S_B_* in Figure 1c, respectively. The experimental THz pulse used in this experiment is shown in Figure 3a. The yellow dot in this figure represents the position of the peak electric field where each THz image was studied in the following demonstrations. Figure 3b,c show the comparative temporal maps at the peak position of the THz electric field without and with probe power normalization, respectively. The four maps in these two sets of figures represent the imaging results using four objectives, 2×, 5×, 10×, and 20×, respectively. From these maps, it is evident that the probe power normalization of the THz image works and completely eliminates speckle-like variation at all magnification conditions. This is even more significant for higher magnification factors, such as 10× and 20×, where small dust particles produce larger probe modulations. To better appreciate this correction, Figure 3d shows the extraction of the beam time profiles using a 5× objective without (black) and with (red) probe power normalization at the positions between the arrows in Figure 3b,c, respectively. Clearly, the recovery of a Gaussian beam profile is achieved with a single pixel line extracted in these images and without any notable increase in noise.

**Figure 4:**
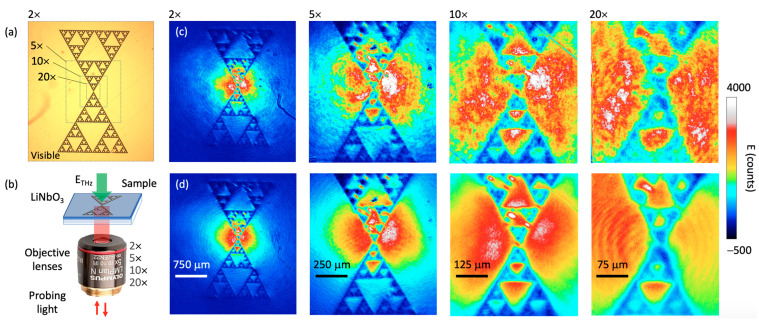
(**a**) Visible image of a fractal metasurface fabricated on a 10 μm thick LiNbO_3_ crystal. The grey dotted areas represent the portions of the sample seen by the different objective lenses. (**b**) Illustration of the optical probing scheme in reflection using multiple objective lenses. The 2D maps in (**c**) represent the THz electric field at the peak position for the 2×, 5×, 10×, and 20× objectives without probe normalization, respectively, whereas the maps in (**d**) show the improvement after applying a background probe normalization. All THz images are composed of a cropped area of 834 × 960 pixels. See also the movies of each of these conditions in the Supplementary Material.

To demonstrate the usefulness of our proposed method, we show the THz imaging evaluation of a fractal sample; see the visible structure in Figure 4a. The sample is patterned directly on the LN sensor, as shown in Figure 4b, and reported previously [13]. Each magnification area visualized by the different objectives is represented by the grey dotted boxes in Figure 4a. It is important to mention the value of using a higher magnification factor, which does not necessarily improve the spatial resolution, but rather, the sensitivity of the measurement. Basically, a higher magnification increases the number of pixels per unit area, but at the cost of a smaller field of view. For example, the 2× lens produces images with a 2.25 × 2.5 mm^2^ field of view for 834 × 960 pixels, which gives 2.7 μm/pixel, whereas a 20× lens gives 0.27 μm/pixel in an area of 225 × 250 μm^2^. As this system is mainly limited in resolution by the condition of the probing light and the thickness of the EO crystal [13], i.e., around 20 μm for this demonstration, the additional pixels can be used to perform spatial binning [20].

Again, it is striking to visually confirm the clarity and flatness of the THz maps after the two-dimensional probe power normalization treatment (see also the full THz movies in Supplementary Material). It is only when using a 20× magnification objective that some fringes appear in the normalized map; see the last image in Figure 4d. A simple explanation for this appearance of fringes is the split propagation in the last part of the probe path before reaching the camera. Indeed, the *S* and *P* polarized images must be separated for polarization analysis; see Figure 1a. The presence of small dust particles in either the *S* or *P* arms will not be normalized during the acquisition process. To be completely immune to such an event, an independent and dynamic normalization of the probe power for both *S* and *P* images could be performed. However, our field programmable gate array (FPGA) program does not allow for easy modification and this will be part of a future work.

## 4. Conclusions

We reported on the two-dimensional EO imaging of THz beams in the near field using a novel probe power normalization. Using 2×, 5×, 10×, and 20× objectives and a fractal-shaped sample, we visually confirmed the acquisition of images with uniform, linear responses and no added noise, which is of particular importance for high magnification images. Coupled with intense THz pulses, these megapixel-sized, spatially enhanced THz images, captured at video rate, will certainly constitute an important asset for the study of nonlinear optics at spatially resolved THz frequencies under diffraction. Finally, our results suggest a dynamic implementation of this two-dimensional probe power normalization procedure.

## Data Availability

Data underlying the results presented in this paper are not publicly available at this time but may be obtained from the authors upon reasonable request.

## Supplementary Materials

The following supporting information can be downloaded at: https://www.mdpi.com/article/10.3390/s22124482/s1, see the movies of Figure 4 conditions in the supplementary material.
